# CRISPR/Cpf1–FOKI-induced gene editing in *Gluconobacter oxydans*

**DOI:** 10.1016/j.synbio.2024.02.009

**Published:** 2024-03-04

**Authors:** Xuyang Wang, Dong Li, Zhijie Qin, Jian Chen, Jingwen Zhou

**Affiliations:** a Engineering Research Center of Ministry of Education on Food Synthetic Biotechnology, Jiangnan University, 1800 Lihu Road, Wuxi, Jiangsu, 214122, China; b Science Center for Future Foods, Jiangnan University, 1800 Lihu Road, Wuxi, Jiangsu, 214122, China; c Key Laboratory of Industrial Biotechnology, Ministry of Education and School of Biotechnology, Jiangnan University, 1800 Lihu Road, Wuxi, Jiangsu, 214122, China; d Jiangsu Province Engineering Research Center of Food Synthetic Biotechnology, Jiangnan University, Wuxi, 214122, China

**Keywords:** Cell growth, CRISPRi, Anti-CRISPR protein, Multi-gene editing, Vitamin C

## Abstract

*Gluconobacter oxydans* is an important Gram-negative industrial microorganism that produces vitamin C and other products due to its efficient membrane-bound dehydrogenase system. Its incomplete oxidation system has many crucial industrial applications. However, it also leads to slow growth and low biomass, requiring further metabolic modification for balancing the cell growth and incomplete oxidation process. As a non-model strain, *G. oxydans* lacks efficient genome editing tools and cannot perform rapid multi-gene editing and complex metabolic network regulation. In the last 15 years, our laboratory attempted to deploy multiple CRISPR/Cas systems in different *G. oxydans* strains and found none of them as functional. In this study, Cpf1-based or dCpf1-based CRISPRi was constructed to explore the targeted binding ability of Cpf1, while Cpf1–FokI was deployed to study its nuclease activity. A study on Cpf1 found that the CRISPR/Cpf1 system could locate the target genes in *G. oxydans* but lacked the nuclease cleavage activity. Therefore, the CRISPR/Cpf1–FokI system based on FokI nuclease was constructed. Single-gene knockout with efficiency up to 100% and double-gene iterative editing were achieved in *G. oxydans*. Using this system, AcrVA6, the anti-CRISPR protein of *G. oxydans* was discovered for the first time, and efficient genome editing was realized.

## 1 Introduction

*Gluconobacter oxydans* is a Gram-negative, strictly aerobic acidophilic bacterium belonging to the family Acetobacteriaceae [1]. *G. oxydans* is known for its extensive incomplete oxidation of sugar alcohols. The bacterium possesses a variety of membrane-bound dehydrogenases that can partially oxidize alcohols and sugars into corresponding sugars or organic acids in the periplasmic space while obtaining energy. The corresponding products (aldehydes, ketones, and organic acids) are almost entirely secreted into the medium [2]. Since the 1930s, *G. oxydans* has been used in the industrial conversion of D-sorbitol into l-sorbose, production of vitamin C, and bioconversion of several other oxides, making it an essential industrial strain to achieve incomplete oxidation [3]. However, *G. oxydans* has the characteristics of slow growth, low biomass accumulation, and complex plasmid transformation. Additionally, as a non-model microorganism, it lacks efficient gene editing tools, making its molecular modification difficult [4].

At present, the genomes of several *G. oxydans* strains have been reported. *G. oxydans* ATCC 621H (163.1, 26.6, 14.6, 13.2, and 2.7 kb), WSH-003 (170 kb), CGMCC1.0110 (213.4 kb), and WSH-004 (132, 14.5, and 165 kb) have five, one, one, and three endogenous plasmids, respectively [5]. These plasmids pose a huge natural burden to the growth and metabolism of *G. oxydans*. Integrating exogenous pathways with plasmids has certain limitations for metabolic modification in this strain. Also, the expression of a single plasmid of about 10 kb or the simultaneous expression of two plasmids imposes a great burden on the growth of *G. oxydans*. Therefore, only the heterologous expression of simple metabolic pathways can be performed. Integrating foreign genes or metabolic pathways into the genome can avoid the metabolic burden based on plasmid expression, ensure stable inheritance, and avoid antibiotic screening; the resulting engineering strains are more suitable for industrial applications [6]. Therefore, developing effective genome editing technology holds great significance for the transformation and modification of *G. oxydans*. Currently, allelic replacement systems based on the sucrose lethal gene *sacB* [7] encoding l-sucrase and *upp* [8,9] encoding uracil phosphoribosyl transferase are still commonly used gene editing tools in non-model strains. However, these methods are not suitable for multi-gene editing and complex metabolic network regulation.

The nuclease-dependent gene editing tools mainly include the first generation of zinc finger nuclease (ZFN) [10], the second generation of transcription activator-like effector nuclease (TALEN) [11], and the third generation of CRISPR system [12]. ZFN and TALEN rely on specific DNA recognition domains to bind target sequences and achieve DNA double strand breaks through dimerization of the Fok1 shear domain. However, both require the design of specific DNA-binding domains for each edit [13]. The CRISPR/Cas system only needs to design gRNAs to guide Cas nucleases to cleave DNA, which has the advantage of simplicity and low cost. Although many advances have been made in the programmable editing of bacterial and eukaryotic genomes, the genome editing in non-model strains, including integration of complex metabolic pathways, precise regulation of gene strength, multiple gene editing, and so forth, still poses a significant challenge [6,14]. Currently, only a few gene editing methods have been reported in *G. oxydans*. Qin et al. [15] developed a CRISPRi system based on the typical endogenous IE type CRISPR/Cas system in *G. oxydans* WSH-003. The endogenous CRISPRi system was used to study the central carbon metabolic pathway of WSH-003. Additionally, a genome editing method based on the reverse selection marker SacB was developed in *G. oxydans* [4]. Using this system, numerous gene editing types, including gene knockout, insertion and replacement, have been successfully implemented in *G. oxydans*, with the gene editing efficiency of about 50%. The aforementioned methods have been able to meet the gene editing needs of *G. oxydans*, but problems, such as high false-positive rate, large differences in efficiency at different sites, and low efficiency of multi-gene editing, still exist.

In this study, CRISPR systems based on Cas9 and Cpf1 were constructed and tested toward developing efficient editing tools for application in *G. oxydans*. The results showed that the CRISPR systems were ineffective. Afterward, the CRISPRi systems based on Cpf1 and dCpf1, and the Cpf1–FokI fusion structure were deployed to further validate the targeted binding ability and nuclease activity of Cpf1. The results indicated that Cpf1 could target binding genes in *G. oxydans*, without nuclease cleavage activity. Subsequently, the CRISPR/Cpf1 system using FokI nuclease activity was constructed to achieve gene knockout, insertion, replacement, and multi-gene editing in *G. oxydans*. The efficiency of single-gene knockout and double-gene editing reached 80%–100% and 27.5%–45%, respectively. Using CRISPR/Cpf1–FokI, AcrVA6, an endogenous anti-CRISPR (acr) protein of *G. oxydans* was discovered for the first time, and efficient genome editing using the CRISPR system in *G. oxydans* was achieve.

## 2 Materials and methods

### 2.1 Strains, plasmids, growth conditions, and reagents

The strains and plasmids used in this study are listed in Tables S1 and S2. *E. coli* JM109 and BL21 were used for plasmid construction and cultured in LB medium (37 °C, 220 rpm). *G. oxydans* was cultured in sorbitol (50 g/L sorbitol, 10 g/L yeast extract) or glucose (20 g/L glucose, 5 g/L yeast extract, 2.5 g/L MgSO_4_ × 7H_2_O, 1 g/L (NH_4_)_2_SO_4_, 1 g/L KH_2_PO_4_, and 10 μM thymidine) medium (30 °C, 220 rpm). Cephalexin, gentamicin, and kanamycin (50 μg/mL) were added to the medium as needed. All chemical reagents were purchased from Sangon Biotech Co., Ltd (Shanghai, China).

### 2.2 Plasmid construction

The primers used for plasmid construction are listed in Table S3. The primer pair F1/R1 was used to linearize the laboratory-stored plasmid pBBR1MCS-5. The fragments Cas9 and Cpf1 were PCR amplified using the primer pairs F2/R2 and F3/R3 with the templates of laboratory-stored SpyCas9 and FnCpf1 synthesized by Sangon Biotech Co., Ltd (Shanghai, China). The promoter P_114_ was PCR amplified using the primer pair F4/R4, with the genomic DNA of *G. oxydans* WSH-003 as the template. The amplified P_114_, Cas9/Cpf1, and linearized pBBR1MCS-5 were assembled using the Gibson assembly kit (NEB, Beijing, China), generating plasmids pBBR1MCS-5-P_114_-Cas9/Cpf1. The primer pair F5/R5 was used to linearize the laboratory-stored plasmid p2-1. The fragment sgRNA scaffold was PCR amplified using the primer pair F6/R6 with the templates of laboratory-stored plasmid pTarget. The promoter leader was PCR amplified using the primer pair F7/R7, with the template of *G. oxydans* WSH-003. The amplified leader, sgRNA scaffold, and linearized p2-1 were assembled using the Gibson assembly kit (NEB, Beijing, China), generating plasmid p2-1-leader-sgRNA. The plasmid p2-1-leader-crRNA was cyclization PCR amplified using the primer pair F8/R8, with the template of plasmid p2-1-leader-sgRNA.

### 2.3 Preparation of competent cells and electroporation methods

Preparation of competent cells and electroporation methods of *G. oxydans* were described by previous reports [15].

### 2.4 The crRNA design

CRISPR RGEN Tools (http://www.rgenome.net/cas-designer/) was used to design crRNA. In the “CRISPR/Cas-derived RNA-guided Endonucleases” option, “FnCpf1 from Francisella: 5′-TTN-3'” and “*Gluconobacter oxydans* (ASM58385v1)” were selected. The target gene sequence limited to 1 kb was submitted. Two crRNAs with a position difference of 40–46 between the template and non-template chains were selected, corresponding to a distance of 14–20 bp on the genome.

### 2.5 Gene editing method

The plasmid pBBR1MCS-5-p114-FnCpf1 was transferred into *G.oxydans* by the above electroporation method, and then the cells carrying the plasmid were prepared into competent cells. Next, 600 ng plasmid pK18mobSacB-leader-crRNA and 1 μg homologous arm fragment were transferred into the above competent cells. The electroporation product was incubated in sorbitol liquid medium containing 50 μg/mL cephalexin and gentamicin for 5 h, then coated on sorbitol agar (50 μg/mL cephalexin, gentamicin and kanamycin) for about 48 h until visible colonies developed.

The editing efficiency was calculated as number of mutants/total colonies selected. 96 single colonies were randomly selected for colony PCR. The experiment was repeated three times and the average value was taken to calculate the editing efficiency.

The plasmid pK18mobSacB expressing crRNA must be cured before the next gene editing. The last gene-edited strain was cultured in liquid sorbitol medium containing 50 μg/mL cephalexin and gentamicin. Then, it was coated on a solid sorbitol plate (50 μg/mL cephalexin and gentamicin), and cultured at 30 °C for 2 d. After that, single colonies were selected and continued to be cultured in liquid sorbitol medium (50 μg/mL cephalexin and gentamicin). The cultures were coated on a solid sorbitol plate (10 g/L sucrose, 50 μg/mL cephalexin and gentamicin) and cultured at 30 °C for 2d, single colonies were selected for PCR verification.

### 2.6 Protein expression and purification

The procedures of protein expression and purification were conducted according to previous reports with slight modifications [16]. FnCpf1 was fused with N-terminal His tag and cloned into pET28a vector. All the synthesized plasmids were independently transformed into *E. coli* BL21(DE3). *E. coli* adding 0.2 mM IPTG induced protein overexpression at 16 °C for 24 h. Then, cells were collected and resuspension in the binding buffer (25 mM Tris-HCl pH 7.5, 500 mM NaCl) was added and decomposed by ultrasonic wave. The protein was purified by Ni-NTA resin and rapid protein liquid chromatography (FPLC) (AKTA Pure, GE Healthcare). After washing with the binding buffer with 10 mM imidazole added, the target protein was eluted with an elution buffer (25 mM Tris-HCl pH 7.5, 500 mM NaCl, 300 mM imidazole).

### 2.7 DNA cleavage assay in vitro

In vitro dsDNA cleavage was performed in a 20 μL system containing 2 μg FnCpf1, 300 ng crRNA and 300 ng dsDNA. The target DNA sequence containing a protospacer target sequence and a 5' -TTA-3′ PAM motif was amplified by PCR and purified for future use. crRNAs were transcribed in vitro using T7 High Yield RNA Transcription kit (Vazyme Biotech Co., LTD, Nanjing, China)) and purified using column RNA purification kit (Sangon Biotech Co., Ltd, Shanghai, China). Purified FnCpf1 proteins and crRNA were first incubated at room temperature for 10 min in cleavage buffer (50 mM Tris-HCl, pH 7.5, 10 mM MgCl_2_, 100 mM NaCl). The dsDNA was then added to the mixture. The lytic reaction was performed at 37 °C for 20 min. Then 40 mM EDTA and 1 mg/mL protease K were added to terminate the reaction. The lysates were separated and observed by 0.5% TBE agarose gel with RedSafe™ Nucleic Acid Staining Solution (INtRON Biotechnology).

### 2.8 Plasmid interference assays in *E. coli*

The plasmids used for interference tests (pCpf1 and pTarget) were designed according to pCas9 (Addgene 42876) and pTarget (Addgene 62226). pCpf1 was built by replacing the Cas9 of pCas9 with FnCpf1. The DNA sequence of acr candidates was encoded by PCR amplification from *G. oxydans* 1.0110, and was linked to Cpf1 of the pCpf1 vector by rbs. *rimL* in *E. coli* BL21 was selected as the target gene.

Plasmid conversion to *E. coli* using the CaCl_2_ heat shock procedure, as described [17]with minor modification. *E. coli* BL21 strain carrying the above pCpf1 plasmid was cultured overnight in LB medium containing 1% arabinose. The bacterial solution was inoculated on fresh LB medium (1% arabinose) at a ratio of 1% and continued to culture for 2 h and the competent cells were prepared. The pTarget plasmid (containing a spacer sequence targeting *rimL*) was then transformed into the competent cells. After incubating in LB medium containing 1% arabinose for 2 h, the cultures were coated on LB agar (50 μg/mL kanamycin, 50 μg/mL streptomycin and 1% arabinose) and incubated at 37 °C for 12 h. The inhibition efficiency of acr candidates was measured by calculating the ratio of wild-type colonies to the total number of selected colonies.

### 2.9 Fluorescence measurement

The fluorescence intensity was tested by the method described by Qin et al. with a slight modification [4]. A single colony of *G. oxydans* carrying CRISPRi plasmid was selected and cultured in 1 mL of sorbitol medium overnight. Then, 100 μL of the seed culture was transferred to 1 mL of sorbitol medium and cultured for 8 h for determining fluorescence intensity. Furthermore, 200 μL of the culture broth was added to a black 96-well plate. The fluorescence intensity of the culture broth was measured using a microplate reader (BioTek, Winooski, VT, USA). The excitation and emission wavelengths of mCherry were 587 nm and 610 nm, respectively.

### 2.10 High-performance liquid chromatography analysis

With some improvements, the protocol used by Kiefler et al. [18], was followed for high-performance liquid chromatography (HPLC) analysis. Pyruvate, succinate, and fumarate concentrations were determined using HPLC LC-20A (Shimadzu, Kyoto, Japan) loaded with an Aminex HPX-87H column (300 × 7.8 mm^2^; Bio-Rad, CA, USA) at 25 °C with 8 mM H_2_SO_4_ as the eluant at a flow rate of 0.6 mL/min. The retention time of pyruvate, succinate, and fumarate was 11.5, 14.6, and 20.42 min, respectively, under the UV detector at 215 nm.

## 3 Results

### 3.1 Construction of CRISPR/Cas9 and CRISPR/Cpf1 systems of *G. oxydans*

**Fig. 1:**
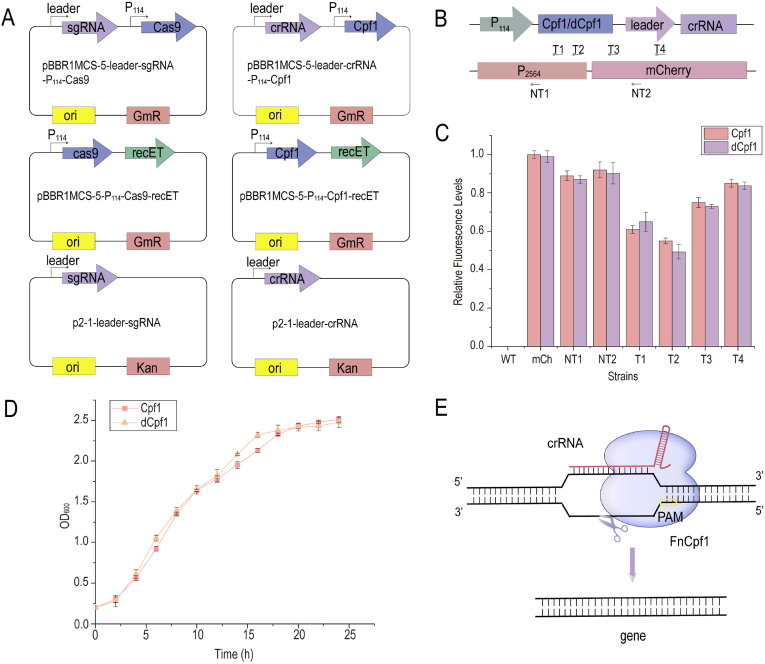
**Construction and verification of CRISPR and CRISPRi systems.** (A) Schematic diagram of CRISPR plasmid construction. The single and double plasmid systems of CRISPR/Cas9 and CRISPR/Cpf1 were constructed respectively. The single plasmid system uses pBBR1MCS-5 as the expression vector, the promoter leader expresses sgRNA or crRNA, and the promoter p114 expresses Cas9 or Cpf1. In the double plasmid system, pBBR1MCS-5 plasmid and p114 promoter express Cas9 or Cpf1, and homologous recombinant protein repET, p2-1 plasmid and promoter leader express sgRNA or crRNA. (B) Illustration of CRISPRi inhibiting *mCherry* expression and different suppressive sites of *mCherry*. T1 and T2 are located downstream of promoter −10 region, while NT1 is located upstream of promoter −35 region. T3, T4, and NT2 are located near the initiation codon of mCherry. T1, T2, T3, and T4 are on the template chain, while NT1 and NT2 are on the non-template chain. (C) Relative fluorescence levels of mcherry after CRISPRi system targets different sites. WT stands for *G. oxydans* CGMCC 1.0110 carrying p2-1, and mch stands for *G. oxydans* CGMCC 1.0110 carrying p2-1-p2564-mCherry. CRISPRi plasmids containing different crRNA were transferred to mch to obtain strains NT1, NT2, T1, T2, T3 and T4. (D) Growth of *G. oxydans* CGMCC 1.0110 carrying with pBBR1MCS-5-p114-Cpf1 and pBBR1MCS-5-p114-dCpf1. (E) Schematic diagram of the CRISPR system in *G. oxydans.* It is speculated that Cpf1 and crRNA complex can bind target DNA in *G. oxydans*, but cannot break DNA. C, D Error bars here represent the standard deviation of three biological replicates (n = 3).

Studies on gene knockout and knock-in using the CRISPR system in *G. oxydans* are lacking. In this study, CRISPR/Cas9 and CRISPR/Cpf1 were deployed in *G. oxydans*. The Cas protein and gRNA were expressed using the broad host plasmid pBBR1MCS-5 and the shuttle plasmid p2-1(1) available in *G. oxydans*, respectively. Furthermore, the single-plasmid system was also constructed (Fig. 1A). Using *gdhM* (glucose dehydrogenase) as the target gene, it was found that the CRISPR/Cas9 system was unable to perform gene knockout against *G. oxydans* in nearly 50 experiments (about 1500 transformants) (Supplementary Fig. 1A). Only one knocked-out strain was obtained using CRISPR/Cpf1 (approximately 5000 transformants) (Supplementary Fig. 1B).

The subsequent optimization of CRISPR/Cpf1 system was expected to improve the editing efficiency of the system, adjust the expression intensity of Cpf1 and crRNA by replacing promoters with different intensity gradients, change the length of homologous arms and increase the supply concentration of homologous arms. In addition, recAR protein of *G. oxydans* and recET protein of *Escherichia coli* were expressed respectively to enhance the homologous recombination ability (Supplementary Fig. 1C). However, the optimized CRISPR/Cpf1 system did not obtain knockout strains again. To test whether the efficiency is affected by the inability of the plasmid to express efficiently in the cell, gene editing is attempted using direct delivery of Cpf1 ribonucleoprotein (RNP). Cpf1 protein was purified (Supplementary Fig. 1D) and crRNA was transcribed in vitro. DNA cleavage experiments showed that Cpf1 RNP had the ability to cut target DNA in vitro (Supplementary Fig. 1E), but the delivery of Cpf1 RNP still failed to achieve gene knockout in the cell. It is assumed that the previous successful knockout strain was an accidental escape, and in fact the CRISPR/Cpf1 system is also ineffective in *G. oxydans*.

### 3.2 Verification of CRISPR/Cpf1 system for targeted binding function

*G. oxydans* is characterized by slow growth and difficult transformation. When Cas9-expressing plasmids were transformed into *G. oxydans*, a few transformants grew on sorbitol plates after 3–4 d, which is not conducive to the study of CRISPR system. After the conversion of Cpf1-expressing plasmids, a significant number of visible colonies could grow on the sorbitol plates within 2 d. Therefore, the next focus was on Cpf1. The CRISPR system had the functions of target gene localization and nuclease cleavage. The function of target gene localization was first investigated to explore why the CRISPR/Cpf1 system did not work in *G. oxydans*.

The CRISPRi systems based on Cpf1 and dCpf1 were constructed and characterized using *mCherry*. Six crRNAs were designed in the promoter and *mCherry* gene regions of the expression vector (Fig. 1B). The results indicated that T1 and T2 sites downstream of the promoter −10 region had the highest inhibitory effect, with a 30%–50% reduction in fluorescence. Additionally, targeting NT1 sites upstream of the promoter −35 region and T3, T4, and NT2 sites close to the initiation codon of *mCherry* also had certain inhibitory effects; also, the targeted template chain sites had a stronger fluorescence attenuation effect compared with the non-template chain sites (Fig. 1C). Both Cpf1 and dCpf1-based CRISPRi systems inhibited the expression of *mCherry* with similar inhibitory efficiency at the same location. Furthermore, no significant difference was observed in the growth of bacteria carrying plasmids expressing Cpf1 and dCpf1 (Fig. 1D). This indicated that the Cpf1 protein expressed in *G. oxydans* still could bind to crRNA and localize to the target gene, but lost the nuclease cleavage activity. Based on the aforementioned results, a hypothesis was proposed that the inability of the CRISPR/Cpf1 system to function might be due to the impaired nuclease cleavage function of Cpf1 in *G. oxydans* (Fig. 1E).

### 3.3 Construction and functional verification of the CRISPR/Cpf1–FokI system

**Fig. 2:**
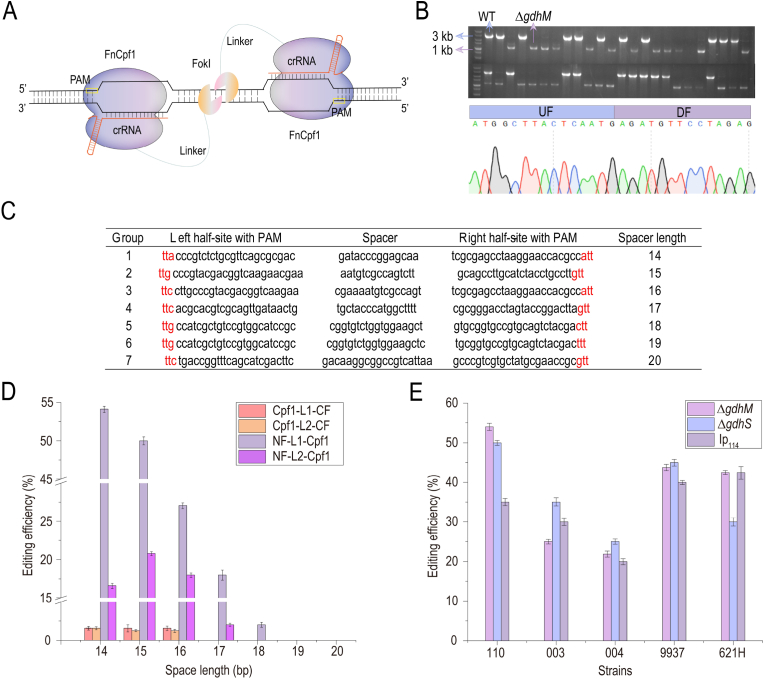
**Construction of CRISPR/Cpf1–FokI system.** (A) Diagram of dimeric Cpf1–FokI. FokI nuclease domains (*orange*) fused to Cpf1 (*purple*) bound to the target DNA with crRNAs (*red*). (B) Knockout of PQQ-dependent membrane-bound glucose dehydrogenase *gdhM*. Above is the gel map of gene knockout, and below is the sequencing results. WT represents the PCR amplification band of wild type strain with a size of 3 kb, and Δ represents the PCR amplification band of gene knockout strain with a size of 1 kb. (C) Multiple groups of crRNAs designed for *gdhM* knockout. Spacer represents the distance between a pair of crRNAs, ranging from 14 to 20 bp. The red bases represent PAM. (D) Deletion efficiency (target *gdhM*) comparison of Cpf1–L1–CF, Cpf1–L2–CF, NF–L1–Cpf1, and NF–L2–Cpf1 mediated by crRNAs at different intervals (14–20 bp). L1 for linker GGGGSx5 and L2 for linker XTEN. CF stands for FokI fusion to the C terminal of Cpf1, and NF stands for FokI fusion to the N terminal of Cpf1. (E) Comparison of the editing efficiency of three genes in different *G. oxydans* strains. Δ*gdhM* represents deletion of the *gdhM*, Δ*gdhS* represents deletion of the *gdhS*, and Ip_114_ represents insertion of promoter p_114_. D, E Error bars here represent the standard deviation of three biological replicates (n = 3).

Based on the aforementioned investigations, it was speculated that CRISPR/Cpf1 system could localize genes in *G. oxydans*, but lacked the nuclease cleavage activity. Therefore, the FokI nuclease was selected to modify the CRISPR system. FokI nuclease is a heterodimer that requires the polymerization of two monomers to perform its cleavage activity (Fig. 2A), so the distance and direction between the two crRNAs were vital. Multiple groups of crRNAs with the orientation of “PAM-out” were designed, where two crRNAs were targeted to a template and non-template chains, respectively, with an interval of 14–20 bp. Two different flexible linkers (L1 for the GGGGSx5 linker of 25 aa and L2 for the XTEN linker of 16 aa) were selected, and FokI was fused to the N and C terminals of Cpf1 using the linker. The knockout strain was successfully obtained by knocking-out the glucose dehydrogenase *gdhM* of *G. oxydans* CGMCC 1.0110 preserved in the laboratory (Fig. 2B).

Among these, NF–L1–Cpf1 formed by connecting FokI to the N terminal of Cpf1 with L1 was a fusion structure with the optimal gene editing effect. Multiple groups of crRNAs with different intervals were tested, the designed crRNA sequences are listed in Fig. 2C, and the interval between the two crRNAs in a group is set from 14 to 20 bp. It was found that gene editing could be detected only when the interval of two crRNAs was 14–18 bp; when the interval was 14–15 bp, the efficiency of cutting target *gdhM* was higher, reaching about 50% (Fig. 2D). Subsequently, the CRISPR/Cpf1–FokI system was constructed with pBBR1MCS-5 expressing NF-L1-Cpf1 and p2-1 expressing two crRNAs at an interval of 14 bp. The knockout of two glucose dehydrogenase genes *gdhM* and *gdhS* and the insertion of the strong promoter p_114_ were carried out in different strains of *G. oxydans* CGMCC 1.0110, ATCC 9937, ATCC 621H, WSH-003, and WSH-004 preserved in the laboratory to verify the generality of the system. The system was found to be generally functional in *G. oxydans* (Fig. 2E).

### 3.4 Development and optimization of the multi-gene editing system

**Fig. 3:**
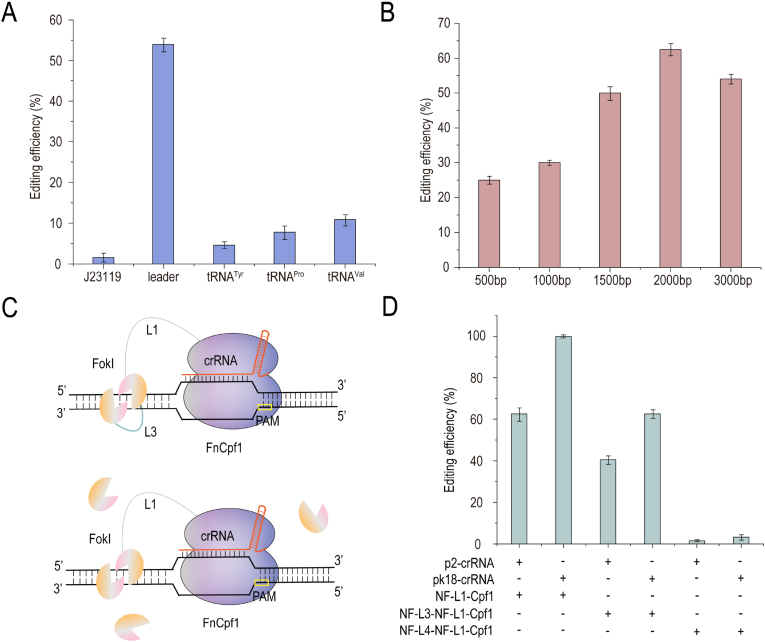
**Development and optimization of single-chain CRISPR/Cpf1–FokI system.** (A) Comparison of *gdhM* deletion efficiency with different RNA promoters. Leader is an endogenous RNA promoter in *G. oxydans* (B) Comparison of *gdhM* deletion efficiency with of different lengths homologous arms. (C) Architectures of single-chain Cpf1–FokI-FokI fusion systems based on a GGGGSx19 linker (L3) or a T2A linker (L4). The first FokI is connected by L1 to the N terminal of Cpf1 (NF-L1-Cpf1), and the second FokI is connected by L3 or L4 to the N terminal of NF-L1-Cpf1. L4 (T2A) is a self-clipping peptide, resulting in the fracture of NF-L4-NF-L1-Cpf1 to form FokI monomer and NF-L1-Cpf1. So L4 is not shown in figure. (D) Comparison of deletion efficiency by NF–L1–Cpf1, NF-L3–NF–L1–Cpf1, and NF–L4–NF–L1–Cpf1 with crRNA expressed by p2-1 and pK18mobSacB plasmids. “+” indicates that the plasmid is present in the strain, and “-” means that the plasmid is not present in the strain. A, B, D data are presented as mean values ± SD from three independent biological replicates (n = 3).

CRISPR array promoter leader of *G. oxydans*, J23119, tyrosine tRNA, valine tRNA, and proline tRNA were compared to further improve the efficiency of the CRISPR/Cpf1–FokI system. Using *gdhM* as the target gene, the results showed that the leader promoter had the best editing effect. The remaining four promoters were extremely inefficient in *G. oxydans* (Fig. 3A). Then, the homologous fragments of 500, 1,000, 1,500, 2,000, and 3000 bp were compared. The efficiency of deleting *gdhM* was found to be the highest when the length of the homologous fragment was 2000 bp (Fig. 3B).

The CRISPR/Cpf1–FokI system requires a pair of crRNAs with a spacing of 14–15 bp to guide the process, which not only increases the difficulty in designing crRNA (it was difficult to find a suitable set of crRNAs for shorter genes), but also is inconvenient for multi-gene editing. Therefore, two CRISPR/Cpf1–FokI–FokI systems were designed to guide Cpf1–FokI–FokI effector proteins with a single crRNA (Fig. 3C). After comparing the editing efficiency of NF–L3–NF–L1–Cpf1 and NF–L4–NF–L1–Cpf1, the long linker (L3: GGGGSx19) was found to be more suitable for constructing Cpf1–FokI–FokI effector protein than the self-clipped peptide (L4: T2A). The probability of contact between the self-clipped FokI monomer and Cpf1–FokI protein in the nucleus was too low to function. Additionally, the crRNA-expressing vector was screened. The results showed that after the pK18mobSacB plasmid expressed crRNA, the knockout efficiency (the target gene is *gdhM*) of the double crRNA-guided NF–L1–Cpf1 system was close to 100%. The knockout efficiency of the NF–L3–NF–L1–Cpf1 system guided by a single crRNA was 62.5% (Fig. 3D).

**Fig. 4:**
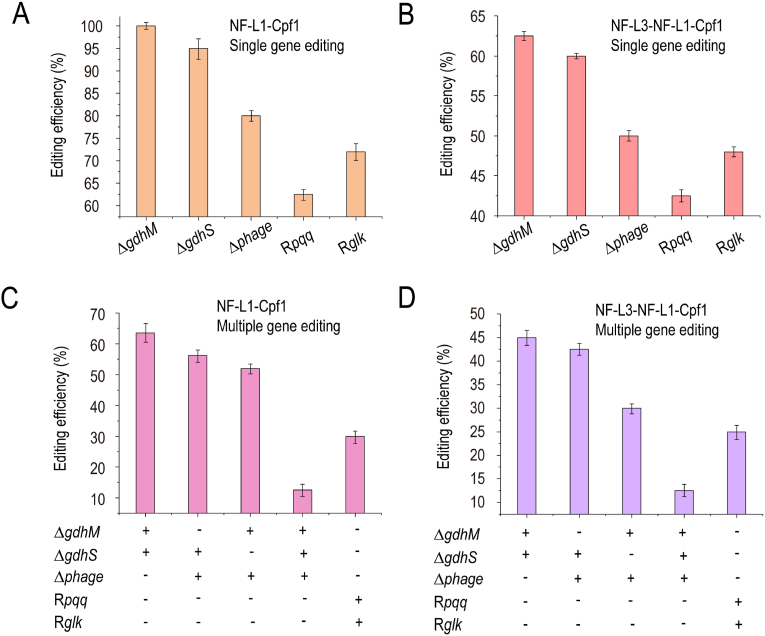
**Evaluation of CRISPR/Cpf1–FokI and CRISPR/Cpf1–FokI–FokI systems.** Efficiency of single-gene editing by NF–L1–Cpf1 (A) and NF-L3-NF-L1-Cpf1 (B). *gdhM*, *gdhS*, and *phage* were selected as knockout genes, and *pqq* and *glk* were selected as replacement genes. The efficiency of multi-gene editing by NF–L1–Cpf1 (C) and NF–L3–NF–L1–Cpf1 (D). The aforementioned five genes were used to validate dual-gene, triple-gene knockout, and dual-gene simultaneous replacement. “**+**” means that this type of gene editing exists, “-” means that it does not exist. A, B, C, D data are presented as mean values ± SD from three independent biological replicates (n = 3).

Three non-essential genes *gdhM*, *gdhS*, and *phage* were selected as knockout genes, *PQQ* gene expression box and *glk* from *Escherichia coli* were selected as replacement genes, and the efficiency of single-gene and multi-gene editing of these two systems was compared. The results showed that the single-gene knockout efficiency of the double crRNA-guided NF–L1–Cpf1 system was 80%–100%, and the single-gene replacement efficiency was 60%–75% (Fig. 4A), double-gene knockout efficiency was 55%–65%, triple-gene knockout efficiency was only 12.5%, and double-gene simultaneous replacement efficiency was 32.5% (Fig. 4C). The efficiency of NF–L3–NF–L1–Cpf1 systems guided by a single crRNA was lower than that of the aforementioned systems, and the efficiency of double-gene editing was 27.5%–45% (Fig. 4D). However, compared with the aforementioned systems, plasmid construction was simpler and more suitable for double-gene editing. Finally, an efficient CRISPR system was constructed, consisting of a pBBR1MCS-5 plasmid carrying p114 promoter for expressing NF–L1–Cpf1 or NF–L3–NF–L1–Cpf1, and a pK18mobSacB plasmid with leader promoter for express crRNA, and 2000-bp donor.

### 3.5 Discovery of the anti-CRISPR (acr) protein AcrVA6 in *G. oxydans*

The functional CRISPR/Cpf1-FokI system supports the previous conjecture that Cpf1 may have lost the DNA-cutting ability of nucleases in *G. oxydans*. Acr protein are CRISPR/Cas antagonists encoded by a variety of mobile genetic elements, such as plasmids and phages, that inhibit CRISPR/Cas immune function at different stages. Firstly, all reported Cpf1 antagonists (AcrVA1, AcrVA2, AcrVA4 and AcrVA5) were searched on NCBI, and these proteins were used as reference sequences for bioinformatics search by BLASTp program. An acetyltransferase with some homology to AcrVA5 was obtained (Supplementary Table S4), but gene editing with wild-type Cpf1 was still not possible after the acetyltransferase was knocked out in *G. oxydan*.

**Fig. 5:**
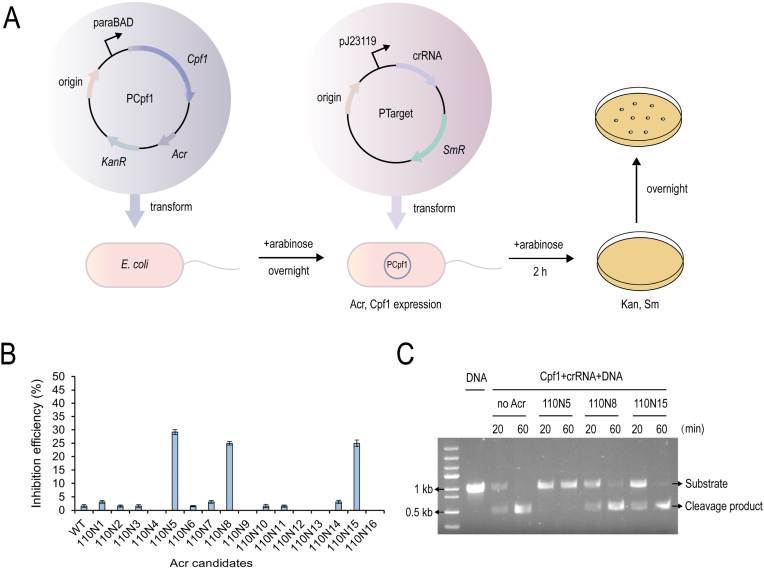
**Identification of anti-CRISPR proteins in *G. oxydans*.** (A) Schematic diagram of plasmid interference assays, including designed plasmids and the experimental procedure in *E. coli*. KanR, kanamycin resistance; SmR, streptomycin resistance. (B) Inhibition efficiency of sixteen acr candidates from *G. oxydans* on CRISPR/Cpf1 system. WT represents the inhibitory efficiency of the CRISPR/Cpf1 system without acr candidates. Error bars represent the standard deviation of three biological replicates (n = 3). (C) Gel images of in vitro DNA cleavage assays in the presence and absence of acr candidates. Representative time-points are shown at the top of each lane. Data shown are representative of three independent experiments.

Given that acr proteins generally show little sequence or structural similarity, and AcrVA5 is an acetyltransferase [16], we expanded our scope to include the sixteen acetyltransferases annotated on the genome of *G*. *oxydans* 1.0110 (Supplementary Table S5). Three acr candidates 110N5, 110N8, and 110N15 were preliminarily screened by the plasmid interference assays in *E. coli* (Fig. 5A). These three proteins had the ability to inhibit Cpf1 gene editing in *E. coli*, with inhibition efficiency of 29.17%, 25%, and 25%, respectively (Fig. 5B, Supplementary Fig. S2). In order to further verify these acr candidates, the three proteins were purified respectively and DNA cleavage experiments were conducted in vitro. The results showed that 110N5 had the ability to inhibit Cpf1 DNA cleavage in vitro (Fig. 5C), which was named AcrVA6. In addition, wild-type Cpf1 showed some gene-editing ability in *G. oxydans* that knocked out AcrVA6. The knockout efficiency was 12.5% (Supplementary Fig. S2E).

### 3.6 Use of CRISPR/Cpf1–FokI system to transform *G. oxydans* to promote growth

**Fig. 6:**
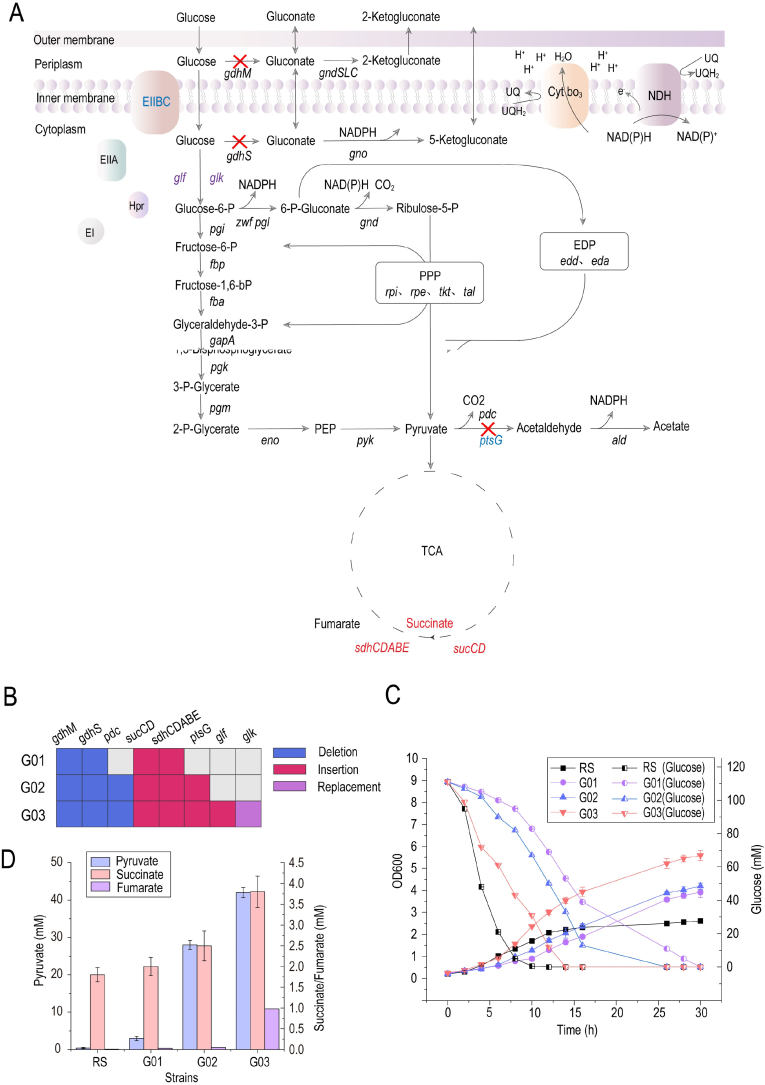
**Engineering glucose metabolic flux in *G. oxydans* for improving growth.** (A) Overall glucose metabolic flow in *G. oxydans* and strategies to increase growth. The red marker genes (*sucCD* and *sdhCDABE*) represent the TCA cycle genes introduced from the other species, the blue marker genes (*ptsG*) denote the PTS genes introduced from the other species, and the purple marker gene (*glf* and *glk*) represents the introduction of glucose transporter genes in the non-PTS. Genes marked with red crosses were deleted during modification. (B) Modified strains constructed in this study. *sucCD*: succinyl-coA synthetase, *sdhCDABE*: succinyl-dehydrogenase, *gdhM*: membrane-bound glucose dehydrogenase, *gdhS*: cytoplasmic glucose dehydrogenase, *pdc*: pyruvate decarboxylase, *ptsG*: EIIBC, *glk*: glucokinase, *glf*: glucose transporter. The growth, glucose consumption (C), and product production (D) in different modified strains. Data are presented as mean values ± SD from three independent biological replicates (n = 3).

*G. oxydans* was characterized by slow growth and low biomass accumulation. Modifying *G. oxydans* to promote growth helped reduce the cost of industrial production and further increase yields [18]. The following strategies were used to promote the growth of *G. oxydans* with glucose as the carbon source: First, the glucose dehydrogenase *gdhM* and *gdhS* were replaced with succinic dehydrogenase *sdhCDABE*_*E. coli*_ and succinyl-CoA synthetase *sucCD*_*E. coli*_ to obtain strain G01. Second, the pyruvate decarboxylase *pdc* of strain G01 was replaced by EIIBC gene *ptsG*_*E. coli*_ to obtain strain G02. Finally, the glucose transporter *glf*_*Z.*__*mobilis*_ and glucokinase *glk*_*E*_. _*coli*_ was integrated into the genome of strain G02 to obtain strain G03 (Fig. 6A and B).

The results showed that the glucose consumption rate of G01 was much slower than the reference strain (RS) after the deletion of glucose dehydrogenase. The RS completely exhausted glucose within 10 h and reached a maximum OD_600_ of 2.5 in 14 h. Although the glucose consumption of G01 was exhausted within 30 h, the OD_600_ of G01 after 30 h was 56% higher than that of RS. At the same time, succinate was detected, indicating the activity of the succinyl-coA synthetase in G01, but the pyruvate content was less improved than that in RS. The glucose consumption rate of G02 was improved to a certain extent after PTS completion to further improve glucose supply. The OD_600_ of G02 in 30 h was 68% higher than that of RS, indicating that PTS completion promoted glucose transport and had a certain promoting effect on the growth of G02. Furthermore, the pyruvate accumulation in G02 significantly increased due to the deletion of pyruvate decarboxylase *pdc*, but the content of succinate and fumarate was still extremely low. After the integration of non-PTS glucose transport pathways, G03 depleted glucose within 14 h and OD_600_ reached 5.9 in 30 h, a 136% improvement over that of RS. The contents of pyruvate, succinate, and fumarate further increased (Fig. 6C and D).

## 4 Discussion

*G. oxydans* lacks efficient genetic manipulation tools, making its metabolic modification difficult. Homologous recombination can only be carried out by transferring homologous fragments [19], with low efficiency and a huge workload. The method based on SacB developed later could assist in operations such as gene knockout and integration. However, SacB usually led to a certain number of false-positive colonies and frequent spontaneous inactivation during the screening process [8,20]. Additionally, the editing efficiency of different genes varied significantly [4]. Therefore, a more efficient, marker-free tool still needs to be developed to achieve complex metabolic modifications in *G. oxydans*.

CRISPR/Cas system is an efficient genome editing tool developed rapidly in recent years; it can achieve multi-gene and large fragment gene editing in different microorganisms [21,22]. We attempted to deploy CRISPR/Cas9, CRISPR/Cpf1, CRISPR/Cas12f [23], CRISPR/Cas12i [24], and CRISPR/Cas12k [6] systems in *G. oxydans*, but none of them could achieve gene editing in the bacterium. Although the CRISPR/Cpf1 system failed to achieve gene knockout, it could achieve gene knockdown in *G. oxydans*. It was speculated that the failure of gene editing in the CRISPR/Cpf1 system might be due to the loss of nuclease cleavage activity. nuclease FokI was fused to the N-terminal of Cpf1 to verify the above conjecture, and it was successfully realized. Because FokI requires the polymerization of two monomers to exert cleavage activity, the distance and direction between the two crRNAs that guide Cpf1-FokI are particularly crucial. A previous study [25] found that when the PAM sequence of the two crRNAs were located at the distal end (“PAM-out” direction) of the target site, they were superior to the proximal end (“PAM-in” direction). Additionally, gene editing was detected for crRNA groups with binding intervals of 14–20 bp for dSpyCas9–FokI and dMbCpf1–FokI [26,27]. This was basically consistent with the results that gene editing was detected at a distance of 14–18 bp between crRNA groups in *G. oxydan*.

Further optimized CRISPR/Cpf1–FokI system could achieve a single-gene knockout efficiency of 80%–100% (Fig. 4A), which was much higher than that of the SacB system (about 50%) [4], and was the most efficient gene editing tool reported in *G. oxydans*. However, as this system required two crRNAs to guide two FokI–Cpf1 monomers to form dimers [25], four crRNAs needed to be expressed simultaneously for double-gene editing. During plasmid construction, two crRNAs could directly obtain by cyclization PCR with a pair of primers. In comparison, Completing four crRNAs in one step requires the Golden Gate Assembly [28], which was more time-consuming and expensive. Therefore, a single crRNA-guided FokI–FokI–Cpf1 system was further developed. Although the editing efficiency of this system was lower than that of the two-crRNA-guided FokI–Cpf1 system, the crRNAs targeting two sites could be directly obtained by cyclization PCR with a pair of primers for dual-gene editing.

Neither the plasmid-expressed CRISPR/Cpf1 system nor the in vitro delivery of Cpf1 RNP could achieve gene editing in *G. oxydans*, while the CRISPR/Cpf1-FokI system fused with FokI nuclease was functional, so Cpf1 antagonists were presumed to exist in *G. oxydans*. CRISPR/Cas antagonists encoded by mobile gene elements, such as plasmids and bacteriophages, were found in *Streptococcus*, *Staphylococcus*, and *Pseudomonas* [29], including AcrIIA family targeting Cas9 [[30], [31], [32]] and AcrVA family inhibiting Cpf1(16). However, further investigations are needed on Acetobacteriaceae as no relevant study exists to date. Dong et al. [16] analyzed fully sequenced bacterial genomes available in the NCBI RefSeq database and did not predict Cpf1-targeting acr candidates in *G. oxydans*. Therefore, this study screened three acr candidates based on BLASTp search and plasmid interference assays in *E. coli*, among which 110N8 and 110N15 did not show significant inhibition effect in in vitro DNA cleavage experiments, although they reduced the editing efficiency of CRISPR/Cpf1 system in plasmid interference assays in *E. coli*. 110N5 (AcrVA6) significantly blocked the cleavage of target DNA by Cpf1. After the elimination of AcrVA6 in *G. oxydans*, wild-type Cpf1 restored certain gene editing ability. But after pcr validation, there were bands representing wild-type and deletion success (Supplementary Fig. S2E), and other acr proteins may still exist.

Given the slow growth and low biomass, the system was used to improve the growth of *G. oxydans* in the glucose medium. The results of whole-genome sequencing showed that the PTS of *G. oxydans* missed the EIIB and EIIC components of the EII protein; however, the associated sugar transporter was still unknown. Additionally, the genome missed several genes for central metabolic enzymes, this resulted in a dysfunctional glycolytic pathway (EMP), TCA, and glyoxylic acid cycle [5] (Fig. 6A). In response to the aforementioned genomic deletion, the strategies to complete the TCA cycle and glucose transport system were used to modify *G. oxydans*. However, the pyruvate content of the strain did not change significantly. This might be due to the insufficient activity of the pyruvate dehydrogenase complex, which prevented further conversion of pyruvate into the downstream pathway. Similarly, Kiefler et al. [18] did not detect pyruvate dehydrogenase complex activity in the cell extracts of *G. oxydans* 621H. Furthermore, the glucose transport capacity further improved after completing the PTS transport system and the *glf/glk* pathway. However, the improvement effect of the non-PTS was more significant than that of the PTS. Similar to that in *E. coli*, the PTS consumed large amounts of PEP, which directly affected the downstream products with PEP as the precursor, such as shikimic acid and aromatic amino acids [33,34]. Conversely, Glf in the *glf/glk* pathway could transport glucose at high speed without any form of energy [35]. *glf*_*Z. mobilis*_ and *glk*_*E. coli*_ were a combination that effectively improved glucose transport rates and the production capacity of compounds with PEP as a precursor [36,37].

In summary, this study developed a CRISPR/Cpf1–FokI system that could be used in *G. oxydans*, and was novel in realizing gene editing with the CRISPR system in *G. oxydans*. The system could realize double-gene iterative editing and had the potential to realize complex genome editing and regulation in *G. oxydans*, and the acr protein AcrVA6 targeting Cpf1 was discovered in *G. oxydans* for the first time. Additionally, this study was another successful case of a tandem combination of tool enzymes, which strongly proved that the cascading tool enzymes with different functions would be the trend of developing complex editing and regulation tools.

## CRediT authorship contribution statement

**Xuyang Wang:** Conceptualization, Methodology, Writing – original draft. **Dong Li:** Validation. **Zhijie Qin:** Validation. **Jian Chen:** Supervision, Funding acquisition. **Jingwen Zhou:** Project administration, Writing – review & editing, Funding acquisition.

## Declaration of competing interest

The authors declare that they have no known competing financial interests or personal relationships that could have appeared to influence the work reported in this paper.
