# Supplementary material for: CRISPR/Cpf1–FOKI-induced gene editing in Gluconobacter oxydans
Source: Synth Syst Biotechnol. 2024 Mar 4;9(2):369–79. doi: 10.1016/j.synbio.2024.02.009 (PMC10980938; doi:10.1016/j.synbio.2024.02.009)
Supplement: Multimedia component 1 [file mmc1.docx]

Supplementary Figures


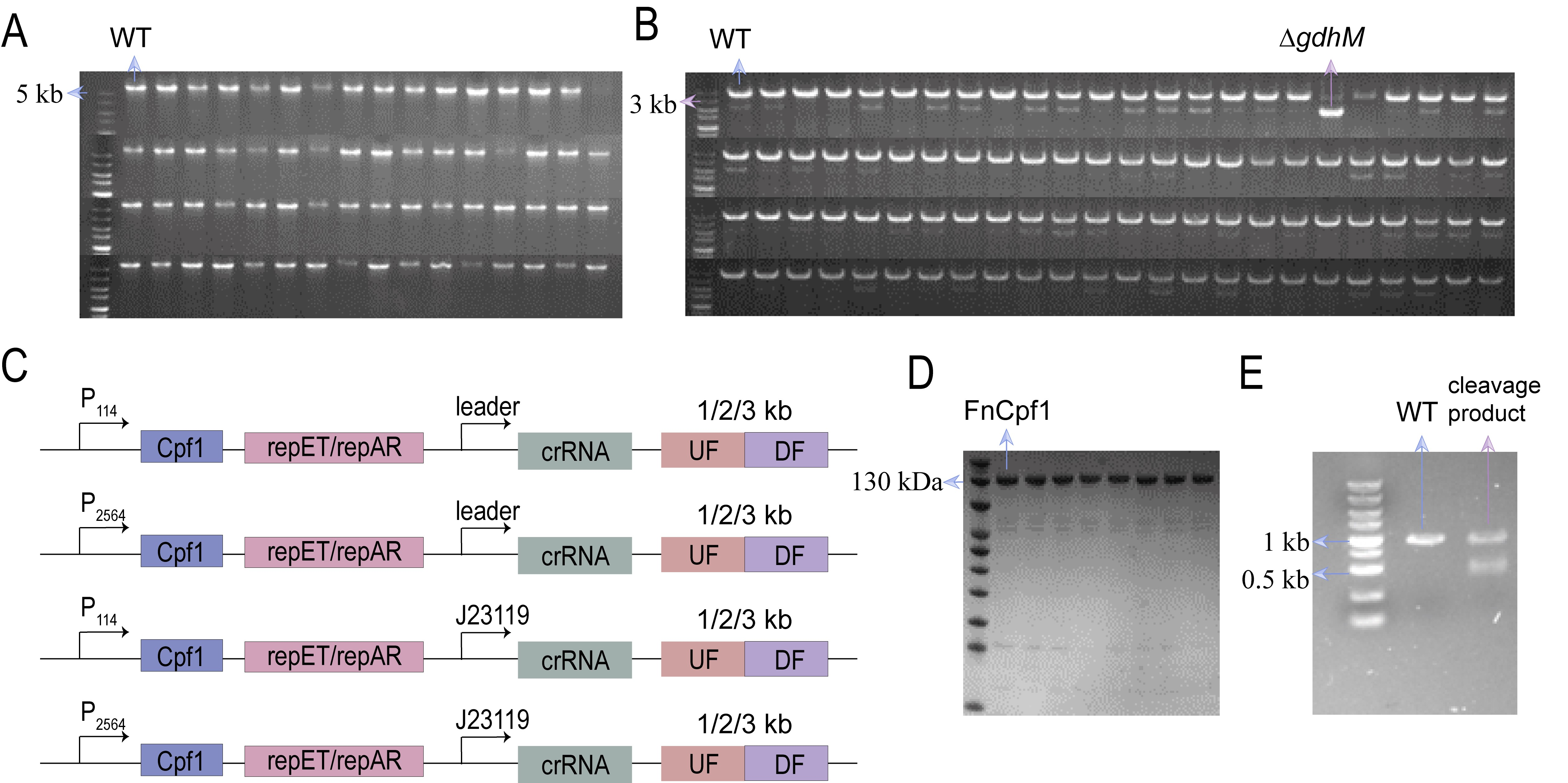


**Supplementary Figure S1. Gene knockout mediated by CRISPR/Cas9 and CRISPR/Cpf1 systems.** (A) Gene knockout guided by CRISPR/Cas9 system. None of the nearly 1,500 transformants obtained in 50 gene-editing experiments were successfully knocked out. Of the nearly 1,500 converters obtained in 50 gene-editing experiments, none were successfully knocked out, and the results of only one gene-editing experiment are shown here. WT was a wild-type strain containing *gdhM*, and marker size was 5 kb. (B) Gene knockout guided by CRISPR/Cpf1 system. Of about 5,000 transformants, there was only one knock-out strain. WT is a wild type strain with a PCR amplification band size of 5 kb. Δ*gdhM* represents a knockout strain with a PCR amplification band size of 3 kb (C) Illustration of a combined optimization CRISPR/Cpf1 system. p_114_ is a strong promoter, while p_2564_ is a moderate promoter. leader is an RNA promoter derived from *G. oxydans*. RepET and repAR are homologous recombinant proteins derived from *E. coli* and *G. oxydans*, respectively. UF represents the upstream homologous arm and DF represents the downstream homologous arm. (D) Protein glue diagram of Cpf1 after purification by nickel column and molecular sieve. The molecular weight of FnCpf1 is 151.9 kDa. (E) DNA cracking gel diagram in vitro. WT is a linear DNA molecule with a size of 1kb, the cleavage product is the mixture of Cpf1, crRNA and DNA incubated, and the cleavage product size is 500 bp.


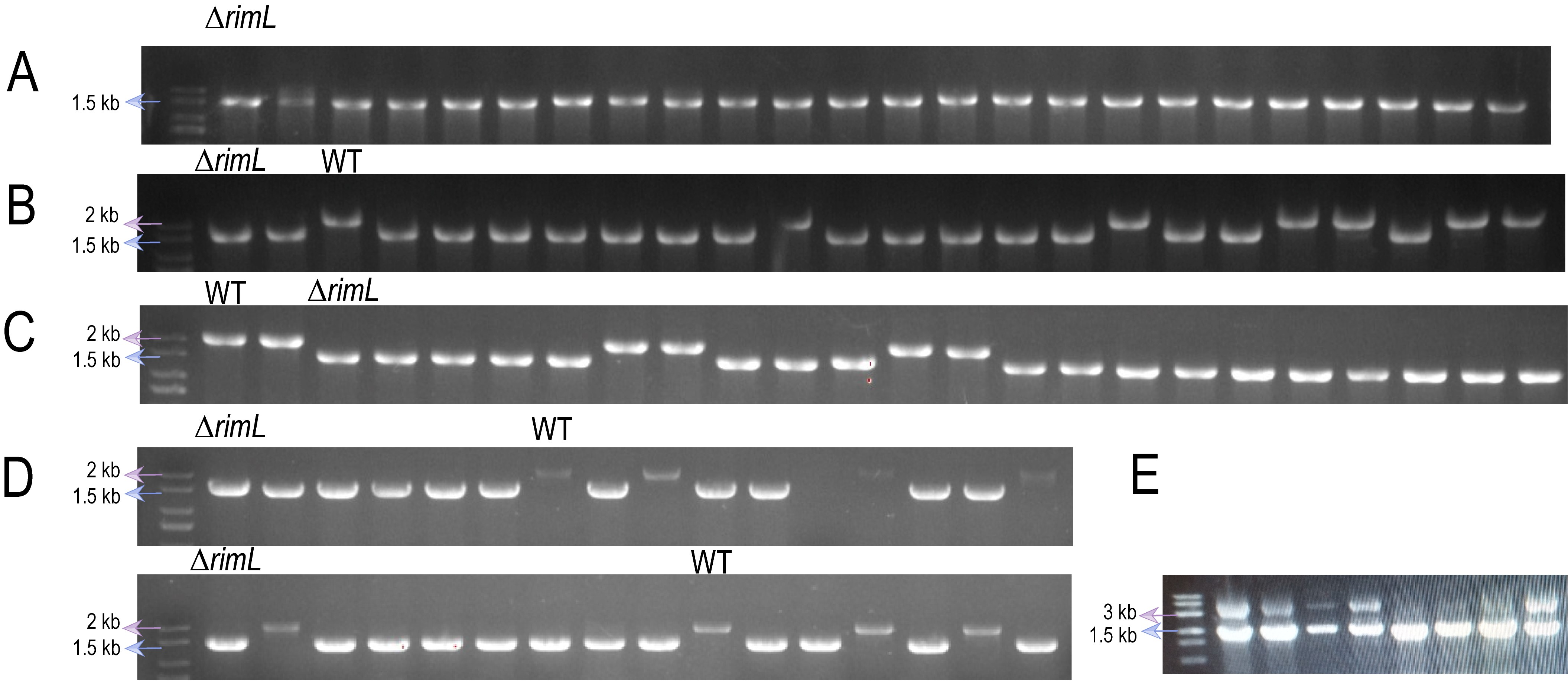


**Supplementary Figure S2. Inhibition effect of Acr candidates on CRISPR/Cpf1 system.** (A) gel images of CRISPR/Cpf1 system knocking out *rimL* without Acr candidates in *E. coli*. (B) gel images of CRISPR/Cpf1 system knocking out *rimL* with 110N5 in *E. coli*. (C) gel images of CRISPR/Cpf1 system knocking out *rimL* with 110N8 in *E. coli*. (D) gel images of CRISPR/Cpf1 system knocking out *rimL* with 110N15 in *E. coli*. *rimL* was selected as the gene for validation of Acr candidates, with a size of 500 bp. WT representing non-knockout and Δ*rimL* representing successful knockout. (E) gel images of CRISPR/Cpf1 system knocking out *gdhM* in *G. oxydans* without 110N5.

**Supplementary Tables**

**Supplementary Table S1 Strains used in this study.**

| Strains | Characteristics | Sources |
| --- | --- | --- |
| 110 | *G. oxydans* CGMCC1.0110 | store |
| 003 | *G. oxydans* WSH-003 | store |
| 004 | *G. oxydans* WSH-004 | store |
| 9937 | *G. oxydans* ATCC9937 | store |
| 621H | *G. oxydans* ATCC621H | store |
| WT | *G. oxydans* CGMCC1.0110 | store |
| mCh | WT with plasmid p2-1-P_2564_-mCherry and pBBR1MCS-5-P_114_-Cpf1/dCpf1 | This study |
| T1 | WT with plasmid p2-1-leader-C_T1_-P_2564_-mCherry and pBBR1MCS-5-P_114_-Cpf1/dCpf1 | This study |
| T2 | WT with plasmid p2-1-leader-C_T2_-P_2564_-mCherry and pBBR1MCS-5-P_114_-Cpf1/dCpf1 | This study |
| T3 | WT with plasmid p2-1-leader-C_T3_-P_2564_-mCherry and pBBR1MCS-5-P_114_-Cpf1/dCpf1 | This study |
| T4 | WT with plasmid p2-1-leader-C_T4_-P_2564_-mCherry and pBBR1MCS-5-P_114_-Cpf1/dCpf1 | This study |
| NT1 | WT with plasmid p2-1-leader-C_NT1_-P_2564_-mCherry and pBBR1MCS-5-P_114_-Cpf1/dCpf1 | This study |
| NT2 | WT with plasmid p2-1-leader-C_NT2_-P_2564_-mCherry and pBBR1MCS-5-P_114_-Cpf1/dCpf1 | This study |
| RS | *G. oxydans* CGMCC1.0110 | store |
| G01 | RS *ΔgdhM::sdhCDABE* *ΔgdhS::sucCD* | This study |
| G02 | G01 *Δpdc::ptsG* | This study |
| G03 | G02 | This study |

**Supplementary Table S2 Plasmids used in this study.**

| Plasmids | Characteristics | Sources |
| --- | --- | --- |
| pBBR1MCS-5 | Wide host vector | store |
| p2-1 | *E. coli-G. oxydans* shuttle vector | store |
| pK18mobSacB | Suicide vector | store |
| pBBR1MCS-5-P_114_-Cas9 | pBBR1MCS-5 with promoter P_114_ to express  Cas9 | This study |
| pBBR1MCS-5-P_114_-Cpf1 | pBBR1MCS-5 with promoter P_114_ to express  Cpf1 | This study |
| p2-1-leader-sgRNA | p2-1 with promoter leader to express sgRNA | This study |
| p2-1-leader-crRNA | p2-1 with promoter leader to express crRNA | This study |
| pBBR1MCS-5-P_114_-Cas9 leader-sgRNA | pBBR1MCS-5 with promoter P_114_ to express Cas9 and promoter leader to express sgRNA | This study |
| pBBR1MCS-5-P_114_-Cpf1 leader-crRNA | BBR1MCS-5 with promoter P_114_ to express Cpf1 and promoter leader to express crRNA | This study |
| P2-1-P_2564_-mCherry | p2-1 with promoter P_2564_ to express mCherry | This study |
| p2-1-leader-C_T1_-P_2564_-mCherry | P2-1-P_2564_-mCherry with crRNA targeting T1 locus | This study |
| p2-1-leader-C_T2_-P_2564_-mCherry | P2-1-P_2564_-mCherry with crRNA targeting T2 locus | This study |
| p2-1-leader-C_T3_-P_2564_-mCherry | P2-1-P_2564_-mCherry with crRNA targeting T3 locus | This study |
| p2-1-leader-C_T4_-P_2564_-mCherry | Pp2-1-P_2564_-mCherry with crRNA targeting T4 locus | This study |
| p2-1-leader-C_NT1_-P_2564_-mCherry | p2-1-P_2564_-mCherry with crRNA targeting NT1 locus | This study |
| p2-1-leader-C_NT2_-P_2564_-mCherry | p2-1-P_2564_-mCherry with crRNA targeting NT2 locus | This study |
| pBBR1MCS-5-P_114_-Fok1-L1-Cpf1 | pBBR1MCS-5 with promoter P_114_ to express Fok1-L1-Cpf1 | This study |
| pBBR1MCS-5-P_114_-Fok1-L2-Cpf1 | pBBR1MCS-5 with promoter P_114_ to express Fok1-L2-Cpf1 | This study |
| pBBR1MCS-5-P_114_- Cpf1-L1-Fok1 | pBBR1MCS-5 with promoter P_114_ to express Cpf1-L1-Fok1 | This study |
| pBBR1MCS-5-P_114_- Cpf1-L2-Fok1 | pBBR1MCS-5 with promoter P_114_ to express Cpf1-L2-Fok1 | This study |
| p2-1-leader-C_14_*_-gdhM_* | p2-1 with promoter leader to express two crRNAs with an interval of 14 bp targeting *gdhM* | This study |
| p2-1-leader-C_15_*_-gdhM_* | p2-1 with promoter leader to express two crRNAs with an interval of 15 bp targeting *gdhM* | This study |
| p2-1-leader-C_16_*_-gdhM_* | p2-1 with promoter leader to express two crRNAs with an interval of 16 bp targeting *gdhM* | This study |
| p2-1-leader-C_17_*_-gdhM_* | p2-1 with promoter leader to express two crRNAs with an interval of 17 bp targeting *gdhM* | This study |
| p2-1-leader-C_18_*_-gdhM_* | p2-1 with promoter leader to express two crRNAs with an interval of 18 bp targeting *gdhM* | This study |
| p2-1-leader-C_19_*_-gdhM_* | p2-1 with promoter leader to express two crRNAs with an interval of 19 bp targeting *gdhM* | This study |
| p2-1-leader-C_20_*_-gdhM_* | p2-1 with promoter leader to express two crRNAs with an interval of 20 bp targeting *gdhM* | This study |
| p2-1-leader-C_14_*_-gdhS_* | p2-1 with promoter leader to express two crRNAs with an interval of 14 bp targeting *gdhS* | This study |
| p2-1-J23119-C_14_*_-gdhM_* | p2-1 with promoter J23119 to express two crRNAs with an interval of 14 bp targeting *gdhM* | This study |
| p2-1-tRNA^Tyr^-C_14_*_-gdhM_* | p2-1 with promoter tRNA^Tyr^ to express two crRNAs with an interval of 14 bp targeting *gdhM* | This study |
| p2-1-tRNA^Pro^-C_14_*_-gdhM_* | p2-1 with promoter tRNA^Pro^ to express two crRNAs with an interval of 14 bp targeting *gdhM* | This study |
| p2-1-tRNA^Val^-C_14_*_-gdhM_* | p2-1 with promoter tRNA^Val^ to express two crRNAs with an interval of 14 bp targeting *gdhM* | This study |
| pK18mobSacB-leader-C_14_*_-gdhM_* | pK18mobSacB with promoter leader to express two crRNAs with an interval of 14 bp targeting *gdhM* | This study |
| pBBR1MCS-5-P_114_-Fok1-L3-Fok1-L1-Cpf1 | pBBR1MCS-5 with promoter P_114_ to express Fok1-L3-Fok1-L1-Cpf1 | This study |
| pBBR1MCS-5-P_114_-Fok1-L4-Fok1-L1-Cpf1 | pBBR1MCS-5 with promoter P_114_ to express Fok1-L4-Fok1-L1-Cpf1 | This study |
| pK18mobSacB-leader-C_14_*_-gdhS_* | pK18mobSacB with promoter leader to express two crRNAs with an interval of 14 bp targeting *gdhS* | This study |
| pK18mobSacB-leader-C_14_*_-phage_* | pK18mobSacB with promoter leader to express two crRNAs with an interval of 14 bp targeting *phage* | This study |
| pK18mobSacB-leader-C_14_*_-glk_* | pK18mobSacB with promoter leader to express two crRNAs with an interval of 14 bp targeting *glk* | This study |
| pK18mobSacB-leader-C*_gdhM_* | pK18mobSacB with promoter leader to express a crRNA targeting *gdhM* | This study |
| pK18mobSacB-leader-C*_gdhS_* | pK18mobSacB with promoter leader to express a crRNA targeting *gdhS* | This study |
| pK18mobSacB-leader-C*_phage_* | pK18mobSacB with promoter leader to express a crRNA targeting *phage* | This study |
| pK18mobSacB-leader-C*_glk_* | pK18mobSacB with promoter leader to express a crRNA targeting *glk* | This study |
| pK18mobSacB-leader-C*_gdhM_*-C*_gdhS_* | pK18mobSacB with promoter leader to express 2 crRNAs targeting *gdhM* and *gdhS* respectively | This study |
| pK18mobSacB-leader-C*_gdhM_*-C*_phage_* | pK18mobSacB with promoter leader to express 2 crRNAs targeting *gdhM* and *phage* respectively | This study |
| pK18mobSacB-leader-C*_gdhS_*-C*_phage_* | pK18mobSacB with promoter leader to express 2 crRNAs targeting *phage* and *gdhS* respectively | This study |
| pK18mobSacB-leader-C*_gdhM_*-C*_gdhS_*-C*_phage_* | pK18mobSacB with promoter leader to express 3 crRNAs targeting *gdhM*, *phage* and *gdhS* respectively | This study |
| pK18mobSacB-leader-C_14_*_-pdc_* | pK18mobSacB with promoter leader to express two crRNAs with an interval of 14 bp targeting *pdc* | This study |

**Supplementary Table S3 Primers used in this study.**

| Primers | Sequence (5'-3') |
| --- | --- |
| F1 | GCCAGAATCAGAAACGGCCC |
| R1 | CCACATCTGCTACCACACTAGTTCTAGAGCGGCCGCCAC |
| F2 | GTTATGAAAGGATTACGCAATGGATAAGAAATACTCAATAGGCTTAGATATCG |
| R2 | CCGTTTCTGATTCTGGCTCAGTCACCTCCTAGCTGACTC |
| F3 | ATGGTCATTTATCAAGAATTTGTTAATAAATATAGTTTAAGTAAAACT |
| R3 | CGGAGTGATCCGGGGCCGTTTCTGATTCTGGCTTAGTTATTCCTATTCTGCACGAACTCAAAAT |
| F4 | TGGTAGCAGATGTGGAGAAATATCCGCAGTTC |
| R4 | TGCGTAATCCTTTCATAACACCATTCGGT |
| F5 | TTGGGTCTGTGGTTCAGAACGGAAGGAC |
| R5 | TTTTACAACGTCGTGACTGGGAAAACCCT |
| F6 | TATAAACTCAAACAACACTGGTTTTAGAGCTAGAAATAGCAAG |
| R6 | CTGAACCACAGACCCAAGACATAAAAAACAAAAAAAGCACCACCGAC |
| F7 | TCACGACGTTGTAAAAGCTCCCAATGACGCAGGCT |
| R7 | TGTTGTTTGAGTTTATACCTTGTTTGTAAGTATATGATTATGGGTGCAAA |
| F8 | ATTCCACTCCCTTCGCTATGAAGGTCCTTTTTTTTGGGTCTGTGGTTCAGAACGG |
| R8 | CGAAGGGAGTGGAATAGATGTTGTCATCTTTAACCTTGTTTGTAAGTATATGATTATGG |
| F9 | TTCATCATTCAATTCAGCACTCACTTGTACAGCTCGTCCATGC |
| R9 | CGAAATCAGGAGTATTTCCCATGGTGAGCAAGGGCGAG |
| F10 | GGGAAATACTCCTGATTTCGTCCTG |
| R10 | TTCTGAACCACAGACCCAAGATACTGTTGTTCGCGCGC |
| F11 | ATTCCACTCCCTTCGCTATGAAGGTCCTTTTTTTTGGGTCTGTGGTTCAGAACGG |
| R11 | TTTTACAACGTCGTGACTGGGAAAACCCTG |
| F12 | TCACGACGTTGTAAAAGCTCCCAATGACGCAGGCT |
| R12 | CGAAGGGAGTGGAATAGATGTTGTCATCTTTAACCTTGTTTGTAAGTATATGATTATGG |
| F13 | ATGGGATCAATGAACGAGGCTTCGCCAGAATCAGAAACGGCC |
| R13 | GAAGCCTCGTTCATTGATCCCATATCTACAACAGTAGAAATTCCTTGTTTGT |
| F14 | AAAAAAATATAAAAAAAGATCCTGCCAGAATCAGAAACGGCCC |
| R14 | GGCAGGATCTTTTTTTATATTTTTTTATCTACAACAGTAGAAATTCCTTGTTTGT |
| F15 | ACATCTAAAAAACTGTTAAATAAGCCAGAATCAGAAACGGCCC |
| R15 | GCTTATTTAACAGTTTTTTAGATGTATCTACAACAGTAGAAATTCCTTGTTTGT |
| F16 | ACCAAGATCCGTAACTGCGTCCCGCCAGAATCAGAAACGGCCC |
| R16 | GGGACGCAGTTACGGATCTTGGTATCTACAACAGTAGAAATTCCTTGTTTGT |
| F17 | GTAAAAGGCATTTCTCTGGATTGGCCAGAATCAGAAACGGCCC |
| R17 | CAATCCAGAGAAATGCCTTTTACATCTACAACAGTAGAAATTCCTTGTTTGT |
| F18 | AGGGCGCCGTCCTCGGGGTACATGCCAGAATCAGAAACGGCCC |
| R18 | ATGTACCCCGAGGACGGCGCCCTATCTACAACAGTAGAAATTCCTTGTTTGT |
| F19 | GTGTTATGAAAGGATTACGCACCTAAGAAGAAGCGCAAGGTCT |
| R19 | CAAATTCTTGATAAATGACCATAGAACCACCACCTCCAGACC |
| F20 | TGGTTCTACCATGGCTCCTAAGAAGAAGCGGAAGGTT |
| R20 | TTGCGCTTCTTCTTAGGGAAGTTGATTTCGCCGTTGTTGAAC |
| F21 | GGTGGTGGTGGTTCTGGAG |
| R21 | AGAACCACCACCTCCAGAC |
| F22 | GGTGGTGGTGGTTCTGGAG |
| R22 | AGCCATGGTAGAACCACCAC |
| F23 | GGTGGTGGTGGTTCTGGAG |
| R23 | AGAACCACCACCTCCAGAC |
| F24 | GAGGGCCGCGGCA |
| R24 | TGGTCCTGGATTCTCCTCCAC |
| F25 | CAGCTGGTCAAGTCCGAACT |
| R25 | TTGCGCTTCTTCTTAGGGAAGTTGATTTCGCCGTTGTTGAAC |
| F26 | ATGGTCATTTATCAAGAATTTGTTAATAAATATAGTTTAAGTAAAACT |
| R26 | TGCGTAATCCTTTCATAACACCATTCGGT |
| F27 | AGCGCGACAATTTCTACTGTTGTAGATCCGCACCAAGGAATCCGAGCGCTTTTTTTTTGGGTCTGTGGTTCAG |
| R27 | TCTACAACAGTAGAAATTGTCGCGCTGAACGCAGAGACGGGATCTACAACAGTAGAAATTCCTTGTTTGT |
| F28 | TTCCATGAATTTCTACTGTTGTAGATTCGATGGCGGTGGGTTCGGGTGGGCCAGAATCAGAAACGGCCC |
| R28 | ACAACAGTAGAAATTCATGGAAGCTCTGTGCGAGGCCGATCTACAACAGTAGAAATTCCTTGTTTGT |
| F29 | ACGGTCAAGAAAATTTCTACTGTTGTAGATCGTCCATCTACGTTCCGACGAAGTTTTTTTTGGGTCTGTGGTTCAGA |
| R29 | TCTACAACAGTAGAAATTTTCTTGACCGTCGTACGGGCAAGATCTACAACAGTAGAAATTCCTTGTTTGT |
| F30 | ATTCAATGAATTTCTACTGTTGTAGATAGCTGGGCGGTGGGATCCGTGACGCCAGAATCAGAAACGGCC |
| R30 | ACAACAGTAGAAATTCATTGAATCCATCGTGAAGGCCAATCTACAACAGTAGAAATTCCTTGTTTGT |
| F31 | CCAGACGGAATTTCTACTGTTGTAGATATGTTCACCGGCAAGAAGTTTGGGCCAGAATCAGAAACGGCCC |
| R31 | ACAGTAGAAATTCCGTCTGGCCTCCGATCCGTGCAATCTACAACAGTAGAAATTCCTTGTTTGT |
| F32 | GGAAATCAATTTCTACTGTTGTAGATGTTCCAGATCCAGACCGGCCAGCGCCAGAATCAGAAACGGCC |
| R32 | ACAACAGTAGAAATTGATTTCCGTGAAGATGATGGCAGATCTACAACAGTAGAAATTCCTTGTTTGT |
| F33 | CGGGTGGAATTTCTACTGTTGTAGATGAAGTAGCGCTCCAGTGCCTGAGGCCAGAATCAGAAACGGCCC |
| R33 | AACAGTAGAAATTCCACCCGAACCCACCGCCATCGAATCTACAACAGTAGAAATTCCTTGTTTGT |
| F34 | ACAGCCAATTTCTACTGTTGTAGATGCGTCCTGCCGCTTCACGATCGAGCCAGAATCAGAAACGGCC |
| R34 | ACAGTAGAAATTGGCTGTTGTCAGCTGGACCAGGAATCTACAACAGTAGAAATTCCTTGTTTGT |
| F35 | gacagctagctcagtcctaggtataatgctagcaatttctactgttgtagatCCCGTCT |
| R35 | gctagcattatacctaggactgagctagctgtcaTTTTACAACGTCGTGACTGGGAAAA |
| F36 | cgctggcgtacgcctacgttggttcgaatccaactctctccaccaaatttctactgttgtagatCCCGTCT |
| R36 | cgtacgccagcggatttacagtccgccccctttagccactcgggcacctctccTTTTACAACGTCGTGACTGGGAAAA |
| F37 | ttgggtacaggaggcccccggttcgagtccgggcgccccgaccaaatttctactgttgtagatCCCGTCT |
| R37 | gcctcctgtacccaaaacaggcgcgctaccaggctgcgccacgccccgTTTTACAACGTCGTGACTGGGAAAA |
| F38 | ttacaccgagagggtcggcagttcaatcctgtcatcgcccaccaaatttctactgttgtagatCCCGTCT |
| R38 | cgaccctctcggtgtaaacgagacgctctaccgctgagctaatcgcccTTTTACAACGTCGTGACTGGGAAAA |
| F39 | ctctagagtcgacctgcaggca |
| R39 | cccgggtaccgagctcgaatt |
| F40 | cgaattcgagctcggtacccgggGCTCCCAATGACGCAGGC |
| R40 | tgcaggtcgactctagagGTGCTGAATTGAATGATGAAACGGC |
| F41 | ACAGCCAATTTCTACTGTTGTAGATGCGTCCTGCCGCTTCACGATCGAGCCAGAATCAGAAACGGCC |
| R41 | ACAGTAGAAATTGGCTGTTGTCAGCTGGACCAGGAATCTACAACAGTAGAAATTCCTTGTTTGT |
| F42 | ATAACTaatttctactgttgtagatAGGCCATGATCCAGGGCGCAAAATTTTTTTTGGGTCTGTGGTTCAGAAC |
| R42 | atctacaacagtagaaattAGTTATCAACTGCGACGTGCGTGatctacaacagtagaaattCCTTGTTTGT |
| F43 | CTGAATaatttctactgttgtagatCACCCTACGCCCGAGGGCGAGGATTTTTTTTGGGTCTGTGGTTCAGAAC |
| R43 | atctacaacagtagaaattATTCAGGCCATGATCCAGGGCGCatctacaacagtagaaattCCTTGTTTGT |
| F44 | AGGAATTTCTACTGTTGTAGATGCATGTTCGAATGGGGTGG |
| R44 | TCTACAACAGTAGAAATTCCTTGTTTGTAAGTATATGATTATGGGTGC |
| F45 | CCGACCGACGATCTTCTTGCCTGGCCAGAATCAGAAACGGCCC |
| R45 | GAAGATCGTCGGTCGGATCTACAACAGTAGAAATTCCTTGTTTGT |
| F46 | TTCTCGACCAGTTGTGTGACAGCGCCAGAATCAGAAACGGCCC |
| R46 | ACACAACTGGTCGAGAAATCTACAACAGTAGAAATTCCTTGTTTGT |
| F47 | GACCGCCCTTTACCAAGGGAAGCGCCAGAATCAGAAACGGCC |
| R47 | TGGTAAAGGGCGGTCATCTACAACAGTAGAAATTCCTTGTTTGT |
| F48 | AGCGCGAATTTCTACTGTTGTAGATACGAAAGCGAAGCGACAGGAGGAGCCAGAATCAGAAACGGCC |
| R48 | CAGTAGAAATTCGCGCTGGACCCTGAAGTTCACGATCTACAACAGTAGAAATTCCTTGTTTGT |
| F49 | GCGTTAATTTCTACTGTTGTAGATGTGGATCATGCGACCGTCGAGCGGCCAGAATCAGAAACGGCC |
| R49 | ACAACAGTAGAAATTAACGCGGACCCGGTCCTCATCCAATCTACAACAGTAGAAATTCCTTGTTTGT |
| F50 | AGGaatttctactgttgtagatACTGTGGCGGCATGGGCTTCATATTTTTTTTGGGTCTGTGGTTCAGAAC |
| R50 | atctacaacagtagaaattCCTCGGGCGTAGGGTGCAACAAAatctacaacagtagaaattCCTTGTTTGT |
| F51 | ACTGaatttctactgttgtagatATTCAGGCCATGATCCAGGGCGCTTTTTTTTGGGTCTGTGGTTCAGAAC |
| R51 | atctacaacagtagaaattCAGTTATCAACTGCGACGTGCGTatctacaacagtagaaattCCTTGTTTGT |

**Supplementary Table S4 Acr candidates searched by BLASTp in *G. oxydans.***

| Acrs | Homologous protein in *G.oxydans* | E-value | Per.Ident | Acession |
| --- | --- | --- | --- | --- |
| AcrIIA1 | hypothetical protein | 0.77 | 50% | GEM17886.1 |
| AcrIIA6 | TonB-dependent receptor | 0.018 | 29.41% | WP_172492731.1 |
| AcrIIA6 | ferric iron siderophore receptor | 0.11 | 29.6% | AFW02990.1 |
| AcrIE | Phage DNA packaging protein | 0.064 | 36.36% | AFW00517.1 |
| AcrVA5 | Putative N-acetyltransferase protein | 0.15 | 26.92% | AAW62119.1 |

Note: Red labeled AcrVA5 is the reference sequence used to identify the anti-CRISPR protein in this study.

**Supplementary Table S5 Amino acid sequences of acr candidates in this study.**

| **Name** | **Amino acid sequences** |
| --- | --- |
| 110N1 | MLPAWLHPQGSSTRFEVGVTFLETRASRPAPVLPEGVSLERVLPPALPLYRKLYREVGEAYCWWMRRPMPDEKLAEIVHDPDVYFMVLWQADGTPLGFYELDLREGGDANLAYFGLLPAAIGHGLGRALLDSAISQAFAAGCWRLRVNTCTLDHPRALTNYQKCGFVIQYVVQEIWNVPDEYVPAELRRL |
| 110N2 | MLGRELRTARLVLTPVNWPDLEDMIALKADAGAFGRMLGGVRSRTEAEQDMAEDVAFWARRRIGIFAIRENGRFVGMTGVHERPDGRGLGLRFSIFPWAAGRGLAREAAGAAIRYVLDEGEPRIVAVAREDNLASRMVLGSIGMRHIDTFKRDGDTMFLYEIAPA |
| 110N3 | MNPNYTLRPADFSEQETRDLLALHLAGMHENTPAGHVFALDLAALTSSDIEVWTVRHRGHVAGIGALKRLRAQDGELKSMRTHPDFLRQGIASTLLDHLIHRARSLGLKKLSLETGQNSSFEPALKLYKTRGFQFGGPFADYQASEFNQFLHLSL |
| 110N4 | MTPELKAGDFLLRPLRPEDAPAVHRLVNDWSVVRMLSRLPFPYPRDLAEKWIASTLEDSARGSAHHFAITRDGVLLGAVGLVLSDDKRSASLGYWASPATWGQGMTTSAARRVTEWAIQVLKLEKLTADVAVDNPASSAVLRKLGFRETGRSSRRFVSRGTECPIIVHELTRSTFLQLDTPAPAETAPSAALEDQPPSPAPTRKPRTLLVVAAALLDAQGRILLARRPEGKRLAGLWEFPGGKVERDETPEQALIREMKEELDVDLTGACLPLSPSSVKMQAHFIF |
| 110N5  (AcrVA6) | MPLYVVRRWRGVPAPREGQALAWVEAADLAKYPMPDPDLPLIPLLQELLG |
| 110N6 | MAKPIPVSPLARPLPDLSTVAGVRLSAVAAGVRYQGRTDLMLAEFVPGTIVAGVYTKNACPGAPILWCRQAQTTPYARALLVNAGNANVFTGRAGIQACEDCADAVSKLLDCPPQDVFLSSTGVIGEKLPQDRIIAALPAAQAGLSEDNWAQAARAIMTTDTFPKAARRDVTIGGKPVRLQGIAKGSGMVAPDMATMLAYVATDAKLPQNVLQSLLASGCAQSFNSITVDSDTSTSDTLMLFATGLADNPAVDDVNDPALAEFTLALNDLLLELALLVVRDGEGATKQIRVEVTGADSNLSAHRIALSIANSPLVKTAIAGEDANWGRVVMAVGKSGEPADRDLLSVAIGGTWIARDGGVVENYDEAPVVAHMKGQDIEITVDMGLDDGQARVWTCDLTHGYIDINGSYRS |
| 110N7 | MTLSLPDLIFQVTGVESAPLLAALHEEAFAGGEVWDADSFTSLLGLPGTEALVVLKGNEPAGFILTRTVMDETEVLTLAVRPSFRRLGLGKSLVEQILPKGKIFLEVSISNNSAKKLYDRCGFIKAGYRRCYYRDGSDALVLVSTPSE |
| 110N8 | MSSTVNLDQLWRNMQAQACSCHDPLIQDVFATNIGDHHSFSSGLASLLGTKLADRSISATAITRLVTSILLEVPGIAESAAEDILAIHDRDPACPDLVTPFLFFKGWQALQVHRIAHHLWQKNRRHLAYHFQSRVNELFAIDIHPAARLGRRLSIDHGTGIVIGETCIIEDDVSLFQDVTLGGTGKLTGNRHPIVRRGAMIGSGAKVLGRLIIGENARIGAASVVLEDIPANATAVGNPARILEPSKVAAAPDAKLPS |
| 110N9 | MDRSVSSFEEDSPVYTHQTMHLEEGMDLECGVHLAPLEVAYCTYGTLAPDRGNAILVCHALTGDQYLAEPNPLTGKPGWWNRMVGPGLPIDTDRFFVICPNVLGGCMGSTGPKSIRVETGRPWDSEFPPITMHDIVASQACLLDHLGIQRLFAVVGGSMGGMQALTWIADYPERVFAALPIATSPFHSAQNIAFNEVSRQAIFADPDWHDGHYRDFDAIPARGLGVARMMAHITYLSEEALSRKFGRKVRSEAPTAVPAASSPSLFGEMFEVESYLRHQGSTFVRRFDANSYLTVTRAMDYFDLAAEHDGDLSAPFRKCETRFCVVSFSSDWLFPTSQSRLLVRALNKAGANVSFVEIESDRGHDAFLLDEPDLDRTVRGFLNGAAEHAGLTKVEGV |
| 110N10- | MRLDQRLIAEMIPTRARVLDVGSGDGTLIDYLYRTRNCDARGIEIDMQSVTQSVAHGLPVMHGDADHDLSYYPDDSFDYVVLQRTLQAVERPREVLRQMLRIGRHAIISFPNFGHWRLRLQLLATGRMPMTSVWSTPWYSTPNIHPCTIRDFLALCEEEGYIVQQWLAIDEDGLKAPWRRSIRFANLFGEQALFLLRRG |
| 110N11 | MAIDILMPALSPTMTEGKLARWLKNEGDTVSAGDVIAEIETDKATMEVEAVDEGILGRILVQEGVEGVAVNTPIAILVEEGEDVPAASSSQAPASVAPADAPAALVNAPPPVTKAAVTTEGSERIFVSPLAKRMAKERGIALASLNGTGPNGRILKRDVEQGGQPATTTPKATPAAPVASEDSVTRVPNSTMRKVIARRLTESKTQVPHFYVSVDIELDALLALRSKLNATAEDNSFKLSVNDMMIKAVGLALKKVQGLNVQFTDSETLHFDNVDISMAVSIPDGLITPIIRNADQKSLREISREAKDLAKRARAGKLKPEEFQGGTFSISNMGMFGVRDFAAIINPPQAGILAIASGEKRAVVRGDQLAIATVMTATLSVDHRAVDGALGAQWLNALRDIVQNPYTLVV |
| 110N12 | MSLENANVREQTAINLRSMTEEDLGAASGLSKVLGWPHRLEDWDFMLRLGHGLVATLESGEIIGTIMWWSFGERHATVGLIIVNGAWQGHGIGTNMLTTVRRLLPGRSISLNATPAGQSLYEKLGFVPTGIVEQHQGNGVSSPIIALDTGQRLRPVVQYDLQDLIAFDRAATGMDRSALVKDLVQNAKGVVIDTEEGIRGYSFCRRFGWGWLIGPLAAETIEQAKALISHWIGTYGGEFIRIDTPTSSGLSGWLDEMGLIRVDVVNTMSTEPVVISENAPQYFALTSQALG |
| 110N13 | MSIVIREVQEADLPAILRITNDAIEHTDALWISTPFTMEQRQKWVADRQAQNFPVFVAVDGDGVVCGYASYGPFRAFEGYAGTVEHSVYVSSECQGRGLGRLLLNRLIQHAKKAGFHVMVAGITAGNEASIALHHRLGFQNNGILPQVGRKGGRWLDLLFMTLCLEAVSKPEKDFP |
| 110N14 | MPHPDAQPPLGRRRHNRPIIDESVNFPAEQATTQFSAFSRTACVARLAAVLIWAFWVSIIQFVLIRLPGSGKIRVPRIFWRMISRILGLQVRIVGTRAGKILNAKDVQRGERPIIYVANHCSWLDIPVVGGTLHTVFVAKGEVGSWPLIGTISRLGRTIFVSRNRQNTGKELQEMTEHLWNGDDIVLFPEGTSTDGSRVLPFLSSFFAVAKPGRLDQAGMPKPPPTLVQPVSIVYDRLEGLPVGRSRRSVFSWYGDMDLTPHLWSFCQWRSMRATILLHPPLDPENFRSRKELANATFKAVSEGAAQLRQGLVPDDQN |
| 110N15 | MSKAPVTPVRDAAALGPYALAVDAMGGDRAPDIVLAGLELAADRHPTARLLLIGDEAVLKPALARYPKAARICDIRHTPASIPMEMKPTAALRVRGSSMRLAMEAVSSGEARGVVSAGNSGAMLALAKIIVKALPGISRPAMVAVQPSARGDTVMLDLGANIACDARNLVEFAVMGEAFAQAALGLPKPTIGLLNVGSEDLKGDERLRQAAERLRDSALAEQFHGFVEGHDITAGTTDVVVTDGFTGNVALKTGEGALKLAFGLLKRVFETNLLTRIGYLLVRPGLERMREWIDPRRYNGAVFVGLNGVVVKSHGGADGEAFASAVDIAMDAVTHNLNDKIRARLDQLGMLGADVASAAASTVAPEPVS |
| 110N16 | MTVVCERVVSSLSPDDMEALCEAVTAGILDGGGFGWLQPPGNQALERYFEGLLLVQERSLYVLREDGIICGAGQLVRPPSSYEAHAATVNLTGFFVAPYARGRGLGRALIQAMIQGAKAMGSTVINCDVRETHAAAIGLFRSLGFEHWGTHPYYARIGGQTVRGLFFSKLLAGEHEAARWQSSIAAPDAGGAPVSPETTKSHQGLMLYPAIDLKDGACVRLRRGEMEDATHYSDDPAAQAKLFEEAGCRHLHVVDLNGAFAGKSTNVPAIESIVKATGLPVQLGGGIRDMAAIERWLEAGVSRIILGSVAVKDPELVRQAARAFPGQIVAGIDARQGRVATEGWAEVSELEANDLALRMEDAGVAAIIFTEITRDGMLAGLDLEQTADMARRLSIPVIASGGVGTLDHLRDLRAIARDVPGISGAIVGRALYDGRISLKDALDVLGSC |

Note: The 110N5 marked in red is the anti-CRISPR protein AcrVA6 identified in this study.
